# Identification and entomopathogenicity of two novel *Sarocladium* species isolated from soil in China

**DOI:** 10.3389/fmicb.2026.1765776

**Published:** 2026-04-10

**Authors:** Wei Chen, Xue Guan, Tongyi Liu, Qiongbo Hu

**Affiliations:** 1 Ganzhou Green Leafy Vegetables Technology Innovation Center, Ganzhou Polytechnic, Ganzhou, China; 2 College of Plant Protection, State Key Lab of Green Pesticide, South China Agricultural University, Guangzhou, China

**Keywords:** biological control, entomopathogenic fungi, green pesticide, microbial pesticide, *Ostrinia furnacalis*

## Abstract

The genus *Sarocladium* has traditionally been associated with plant endophytism and phytopathogenicity, and entomopathogenic activity has not previously been reported in this genus. In this study, two fungal strains isolated from soil samples collected in China were examined using morphological characterization and phylogenetic analyses to determine their taxonomic positions. Their insecticidal activity was further evaluated against third-instar larvae of Ostrinia furnacalis through laboratory bioassays. The two isolates were identified as two novel species, *Sarocladium poyangense* and *S. jiangsuense*, both of which formed distinct clades separate from their closest known relative, *S. kiliense*. Bioassays showed that both species were strongly pathogenic to *O. furnacalis*. Among them, *S. poyangense* exhibited higher virulence, causing 80% larval mortality at a spore concentration of 1 × 10^8^ spores/mL, with an LC₅₀ of 3.13 × 10^7^ spores/mL and an LT₅₀ of 6.08 days. These findings expand the known ecological role of *Sarocladium* by demonstrating, for the first time, its entomopathogenic potential, and suggest that this genus may represent a promising resource for the development of biological control agents against insect pests.

## 1 Introduction

Fungal biodiversity plays a crucial role in ecosystem stability, with many species exhibiting significant potential in agriculture, medicine, and biotechnology (Retter et al., 2024; Shruthi et al., 2020). The genus *Sarocladium* (Hypocreales, Hypocreaceae) is a diverse group of filamentous fungi, widely distributed in aquatic, plant and soil environments. Taxonomically, *Sarocladium* comprises acremonium-like fungi whose species boundaries have been increasingly clarified through the 26 integration of morphological characters and multilocus phylogenetic inference, thereby improving 27 resolutions among closely related lineages and facilitating accurate species delimitation. Morphologically, members of the genus are typically characterized by hyaline septate hyphae, simple phialidic conidiogenous cells, and hyaline conidia that often accumulate in slimy masses, reflecting their adaptation to diverse substrates and microhabitats (Anjos et al., 2020). Some species of *Sarocladium* are known as endophytes, while others are plant pathogens or opportunistic human pathogens (Blaszczyk et al., 2021). Notably, *S. oryzae* is a well-documented phytopathogen responsible for sheath rot disease in rice, whereas *S. strictum* and *S. kiliense* have been isolated from clinical and environmental samples (Choudhary et al., 2022; Ou et al., 2020; Ranjbar-Mobarake et al., 2021). Despite the increasing interest in this genus, its potential as an entomopathogenic fungal group remains largely unexplored (Najamuddin et al., 2024).

Entomopathogenic fungi (EPF) have garnered significant attention in recent decades due to their potential as environmentally friendly biopesticides. These fungi naturally infect and regulate insect populations, making them valuable alternatives to chemical pesticides (Bamisile et al., 2021). Unlike chemical agents, EPF act by penetrating the host, colonizing it, and producing toxins, leading to insect mortality. Some well-known EPF genera, such as *Beauveria*, *Metarhizium*, and *Cordyceps*, have been extensively studied and commercially developed as biocontrol agents (Hong et al., 2024; Sousa et al., 2023). However, the search for novel EPF remains a priority in microbial pest control, as emerging fungal species may offer improved efficacy, host specificity, or resilience in diverse environmental conditions.

Biological control has become an essential component of integrated pest management (IPM) strategies aimed at reducing the negative impact of chemical pesticides (Sani et al., 2020). The overuse of synthetic insecticides has led to serious issues, including pesticide resistance, environmental contamination, and nontarget organism toxicity (Majeed et al., 2025). Biocontrol agents, particularly EPF, provide a sustainable alternative by leveraging natural microbial diversity to suppress pest populations. Fungi capable of infecting insect pests often produce bioactive secondary metabolites with insecticidal properties, further enhancing their efficacy (Litwin et al., 2020; Pedrini, 2022). Thus, identifying and characterizing novel fungal species with insecticidal activity is critical for expanding the biocontrol arsenal and mitigating agricultural pest challenges.

In this study, we report the discovery of two novel *Sarocladium* species isolated from soil samples collected in eastern China. Through morphological examination and multilocus phylogenetic analyses, these isolates were identified as distinct species within the *Sarocladium* genus. More importantly, this study presents the first report of insecticidal activity in *Sarocladium*, revealing its potential as a biocontrol agent. Bioassays against *O. furnacalis* demonstrated significant pathogenicity, highlighting their applicability in pest management. The findings expand our understanding of the ecological role of *Sarocladium* and provide new insights into its potential use in sustainable agriculture.

## 2 Materials and methods

### 2.1 Soil sample collection

Soil samples were collected from the eastern region of China (Shangrao, Jiangxi and Zhenjiang, Jiangsu, June 2023) using a five-point sampling method (Niu et al., 2019). At each collection site, surface soil (10–20 cm depth) was sampled, with approximately 100 g of soil placed in a self-sealing bag. The geographic coordinates, specific location details, and vegetation type at each sampling point were recorded. All samples were stored at 4 °C until further analysis (Chen et al., 2024).

### 2.2 Fungal isolation and culture

*Sarocladium* strains were isolated from the soil samples using the Chen’s method (Chen et al., 2021). The collected soil samples were sieved through a 0.45 mm mesh to isolate fine soil particles. Two subsamples (10 g each) were taken and suspended in 100 mL of 0.1% Tween-80 solution. The mixture was vortexed to homogenize, then left to settle for 10 min to prepare the soil suspension. Subsequently, 100 μL of the suspension was inoculated onto selective media, evenly spread, and replicated three times for each treatment. The plates were incubated at 26 ± 1 °C in a constant temperature incubator until single colonies emerged on the selective medium (potato 200 g, agar 20 g, glucose 20 g, cycloheximide 50 mg, 3,4,5,6-tetrachlorofluorescein 50 mg, chloramphenicol 50 mg, distilled water 1 L; Chen et al., 2021). Individual colonies were then transferred to potato dextrose agar (PDA) medium (potato 200 g, agar 20 g, glucose 20 g, distilled water 1 L) using an inoculating loop for pure culture isolation. This process was repeated until purified isolates were obtained. The purified *Sarocladium* strains were preserved in 20% glycerol solution and stored at −80 °C and subcultured once per year for strain maintenance.

### 2.3 Morphological observations

The purified fungal strains were stamped to obtain 5 mm diameter plugs, which were then inoculated onto PDA, Czapek’s (sodium nitrate 3.0 g, dipotassium hydrogen phosphate 1.0 g, potassium chloride 0.5 g, magnesium sulfate heptahydrate 0.5 g, ferrous sulfate heptahydrate 0.01 g, sucrose 30.0 g, agar 15.0 g, distilled water 1 L), MEA (malt extract powder 130.0 g, agar 15.0 g, chloramphenicol 0.1 g, distilled water 1 L), CYA (yeast extract 5.0 g, sodium nitrate 3.0 g, dipotassium hydrogen phosphate 1.0 g, potassium chloride 0.5 g, magnesium sulfate heptahydrate 0.5 g, ferrous sulfate heptahydrate 0.01 g, sucrose 30.0 g, agar 15.0 g, distilled water 1 L), and DG18 agar plates (anhydrous glucose 10.0 g, casein peptone 5.0 g, agar 15.0 g, monopotassium phosphate 1.0 g, magnesium sulfate 0.5 g, chloramphenicol 0.1 g, nitrofurazone 0.002 g, distilled water 1 L). The plates were incubated at 26 ± 1 °C for 7 days, after which colony size and morphological characteristics were observed and recorded.

Microstructural imaging was performed using both an optical microscope and a scanning electron microscope (SEM). For SEM sample preparation, 5 mm-diameter fungal plugs were inoculated onto 97 PDA plates, with sterilized aluminum foil placed around them. The plates were incubated in the dark at 26 ± 1 °C until the mycelium covered approximately two-thirds of the aluminum foil. The aluminum foil pieces, along with the attached mycelia and spores, were then fixed in electron microscopy fixative. Following dehydration, drying, and gold sputtering, the samples were examined and photographed using SEM.

### 2.4 DNA extraction, PCR, and sequencing

Purified fungal strains were inoculated onto PDA plates and incubated at 26 ± 1 °C for 7–14 days. Specimens and axenic cultures were prepared for DNA extraction. Genomic DNA was extracted using the HP Fungal DNA Mini Kit D3195-02 (Omega Bio-Tek, Georgia, United States) following the manufacturer’s protocol, with slight modifications.

The ribosomal internal transcribed spacer (ITS) region, actin (act1), the large ribosomal subunit (nrLSU), translation elongation factor 1-alpha (tef1), and the RNA polymerase II second largest subunit (RPB2) were amplified using the following primer pairs: ITS1 (TCCGTAGGTGAACCTGCGG)/ITS4 (TCCTCCGCTTATTGATATGC), act1-F (TAATCAGTCACATGGAGGGT)/act1-R (AACCACCGATCCAGACAGAGT; Groenewald et al., 2013), LR0R (GTACCCGCTGAACTTAAGC)/LR5 (ATCCTGAGGGAAACTTC; Vilgalys and Hester, 1990), EF983F (AGTTCGAGGCTGG-TATCTCC)/EF2218R (CCTTGACGGAGACGTTCTT), and RPB2-5Feur (GAYGAYCGKGAYCAYTTCGG)/RPB2-7CReur (CCCATRGCYTGYT-TRCCCAT; Castlebury et al., 2004).

The PCR reaction was carried out in a 25 μL reaction mixture containing 22 μL of 1.1 × S4 Fidelity PCR Mix (Genesand Biotech Co., Ltd., Beijing, China), 1 μL of each forward and reverse primer, and 1 μL of DNA template. The thermal cycling conditions were as follows: initial denaturation at 95 °C for 5 min, followed by 35 cycles of denaturation at 95 °C for 30 s, annealing at 56 °C for 45 s, and extension at 72 °C for 1 min, with a final extension at 72 °C for 10 min. PCR products were verified by 1% agarose gel electrophoresis, and those exhibiting clear single bands were submitted to YouKang Biotech (Guangzhou, China) for Sanger sequencing. The obtained sequences were deposited in the NCBI GenBank nucleotide database, and the corresponding GenBank accession numbers were recorded.

### 2.5 Phylogenetic analyses

Phylogenetic analyses were conducted based on five gene loci: ITS, nrLSU, act1, tef1, and RPB2. Sequences of related fungal type species were retrieved from GenBank and integrated with those obtained in this study. Taxonomic details and corresponding GenBank accession numbers are provided in Table 1. *Chlamydocillium curvulum* CBS 430.66 was designated as the outgroup for phylogenetic tree construction.

**Table 1:** Specimen information and GenBank accession numbers for sequences used in this study.

Species	Voucher information	GenBank accession numbers				
		ITS	*nrLSU*	*tef1*	*RPB2*	*act1*
*S. agarici*	CBS 113717	OQ429828	OQ430089	OQ471158	OQ454227	–
	CBS 126941	OQ429829	OQ430090	OQ471159	OQ454228	–
*S. attenuatum*	CBS 399.73	OQ429830	OQ430091	OQ471160	OQ454229	HG964979
*S. bacillisporum*	CBS 787.69	OQ429831	OQ430092	OQ471161	OQ454230	HG964955
	CBS 425.67	OQ429832	OR052117	OQ471162	OQ454231	HE608633
*S. bactrocephalum*	CBS 749.69	OQ429833	HQ231994	OQ471163	OQ454232	HG964956
*S. bifurcatum*	CBS 137658	HG965009	HG965057	–	–	HG964959
*S. brachiariae*	CGMCC 2192	EU880834	KP715271	–	–	–
*S. citri*	CBS 145044	OQ429834	OQ430093	OQ471164	OQ454233	–
*S. dejongiae*	CBS 699.93	OQ429835	OQ430094	OQ471165	OQ454234	–
	CBS 144929	MK069419	MK069415	–	–	–
	CBS 694.93	OQ429836	OQ430095	OQ471166	OQ454235	–
*S. ferrugineum*	CBS 102673	OQ429837	OQ430096	OQ471167	OQ454236	–
*S. fuscum*	CBS 334.80	OQ429838	OQ430097	OQ471168	OQ454237	–
*S. gamsii*	CBS 707.73	OQ429839	HG965063	OQ471169	OQ454238	HG964965
	CBS 425.73	OQ429840	OQ430098	OQ471170	OQ451838	HG964964
*S. glaucum*	CBS 796.69	OQ429841	HE608657	OQ471304	OQ451839	HE608631
*S. hominis*	CBS 137659	HG965012	HG965060	–	–	HG964962
*S. junci*	CBS 148277	OK664734	OK663773	–	OK651170	–
*S. kiliense*	CBS 122.29	AJ621775	HQ232052	OQ471172	OQ454241	HG964975
	CBS 400.52	OQ429844	KM231729	OQ471174	OQ454243	–
	CBS 289.70	OQ429845	OQ430100	OQ471175	OQ454244	–
	CBS 129746	–	MH876842	OQ471173	OQ454242	–
*S. ochraceum*	CBS 428.67	OQ429846	HQ232070	OQ471176	OQ454245	HG964977
*S. oryzae*	CBS 180.74	OQ429847	OQ430101	OQ471177	OQ454246	HG964978
*S. pseudostrictum*	CBS 137660	HG965029	HG965073	OQ471178	–	HG964981
*S. spinificis*	CBS 170.89	OQ429849	OQ430103	OQ471180	OQ454248	–
	CBS 102676	OQ429848	OQ430102	OQ471179	OQ454247	–
	BCRC 34941	–	JQ954463	–	–	–
*S. strictum*	CBS 334.77	OQ429852	OQ430106	OQ471183	OQ454251	–
	CBS 102039	OQ429850	OQ430104	OQ471181	OQ454249	–
	CBS 346.70	OQ429853	HQ232141	OQ471184	OQ454252	HG964982
	CBS 152.72	OQ429851	OQ430105	OQ471182	OQ454250	–
	CBS 405.80	OQ429854	OQ430107	OQ471185	–	–
*S. subulatum*	MUCL 9939	HG965031	HG965075	–	–	HG964984
*S. summerbellii*	CBS 430.70	OQ429855	OR052118	OQ471186	OQ454253	HG964987
*S. theobromae*	CBS 113440	OQ429856	OQ430108	OQ471188	OQ454255	–
*S. zeae*	CBS 801.69	OQ429858	HQ232152	OQ471190	OQ454257	–
	CBS 800.69	OQ429857	OQ430109	OQ471189	OQ454256	–
*S. clematidis*	MFLUCC 172150	–	–	MN628625	MN628627	–
*S. graminicola*	Pan64	–	MT196391	–	–	MT210533
*S. liquanensis*	ATCC 39306	MF987659	MF987651	–	–	MF987663
*S. mali*	ATCC39307	MF987662	MF987653	–	–	MF987665
	ATCC39308	MF987661	MF987652	–	–	MF987664
*S. mycophilum*	CBS 166.92	MH862348	MH874015	–	–	HG964976
*S. pseudokiliense*	GUM 1905	–	–	OP351542	–	–
*S. sasijaorum*	CBS 147213	NR_173024	MW883839	MW890116	–	MW890032
*S. sparsum*	BCRC FU31121	NR_165865	–	–	–	–
*S. spirale*	BCRC FU31117	NR_165864	–	–	–	–
*S. poyangense*	GDMCC 3.1075	PP863315	PP930916	PV294978	PV294977	PV294976
	ZJ19A01	PP863314	PV248877	PV294981	PV294980	PV294979
*S. jiangsuense*	GDMCC 3.1076	PP863316	PP930917	PV294972	PV294971	PV294970
	JS07C02	PP863317	PV248878	PV294975	PV294974	PV294973
*C. curvulum*	CBS 430.66	OQ429520	OQ055431	–	OQ560709	HE608630

Sequences that have not been sequenced are indicated by “–.”

Multiple sequence alignment (MSA) was performed using MAFFT v7 (https://mafft.cbrc.jp/alignment/server/, accessed on 10 January 2024) with the FFT-NS-i iterative refinement method. The alignments were manually checked and refined using MEGA v7.0. Phylogenetic analyses were carried out using PhyloSuite v1.2.3, employing both Maximum Likelihood (ML) and Bayesian Inference (BI) methods (Zhang et al., 2020). Poorly aligned regions were removed using Gblocks v0.91b, and the optimal partitioning schemes and evolutionary models for both IQ-TREE and MrBayes were determined using ModelFinder v2.2.0. The ML phylogenetic tree was constructed with IQ-TREE v2.2.0, using 1,000 bootstrap replicates to assess branch support. BI analysis was performed using MrBayes v3.2.7a, running Markov Chain Monte Carlo (MCMC) for 2,000,000 generations, sampling every 1,000 generations, with the first 25% of samples discarded as burn-in. The resulting ML and BI phylogenetic trees were visualized using MEGA v7.0 and FigTree v1.4.3.

### 2.6 Virulence assay of *Sarocladium* isolates

Third-instar larvae of *Ostrinia furnacalis* provided by associate researcher Qunfang Weng from the College of Plant Protection at South China Agricultural University, was fed with a special feed (43 g of soybean powder, 43 g of corn flour, 26 g of glucose, 26 g of yeast extract, 1.5 g of sorbic acid, 1.5 g of compound vitamins, 5.7 g of agar, and 358 g of water.) in a constant temperature incubator at 25 ± 1 °C.

The bioactivity of *Sarocladium* strains was evaluated following a modified version of the Chinese agricultural industry standard NY/T 1154.6–2006. The fungal strains were cultured on PDA medium for 7–14 days to induce sporulation. Once sufficient sporulation was achieved, conidia were harvested from colony surfaces and suspended in a 0.1% Tween-80 solution to prepare spore suspensions at concentrations of 1 × 10^8^, 1 × 10^7^, 1 × 10^6^, and 1 × 10^5^ spores/mL. To ensure inoculum quality, conidial viability was assessed by a germination test on PDA, and only suspensions with high germination rates were used for bioassays.

Third-instar larvae of *O. furnacalis* were placed in sterilized centrifuge tubes, immersed in the spore suspension, and gently agitated by inverting the tubes 10 times before transferring the larvae into disposable plastic containers. The larvae were incubated at 25 ± 1 °C and fed fresh feed, which was replaced every 2 days. Mortality was recorded, with larvae considered dead when they showed no response to gentle probing and deceased larvae were transferred to Petri dishes. To confirm fungal infection as the cause of death, fungal hyphae growing on cadavers were reisolated and cultured on PDA medium, fulfilling Koch’s postulates. Control larvae were treated with 0.1% Tween-80 solution under the same conditions. Each treatment included 10 larvae, with three replicates. LC₅₀ and LT₅₀ were estimated using dose–response and time–mortality analyses.

### 2.7 Data analysis

Statistical analyses were conducted using DPS 9.5 (Data Processing System, Zhejiang, China), Excel 2010 (Microsoft, Washington, DC, United States) and SPSS 25.0 (IBM, New York, United States).

## 3 Results

### 3.1 Morphological features

**Figure 1:**
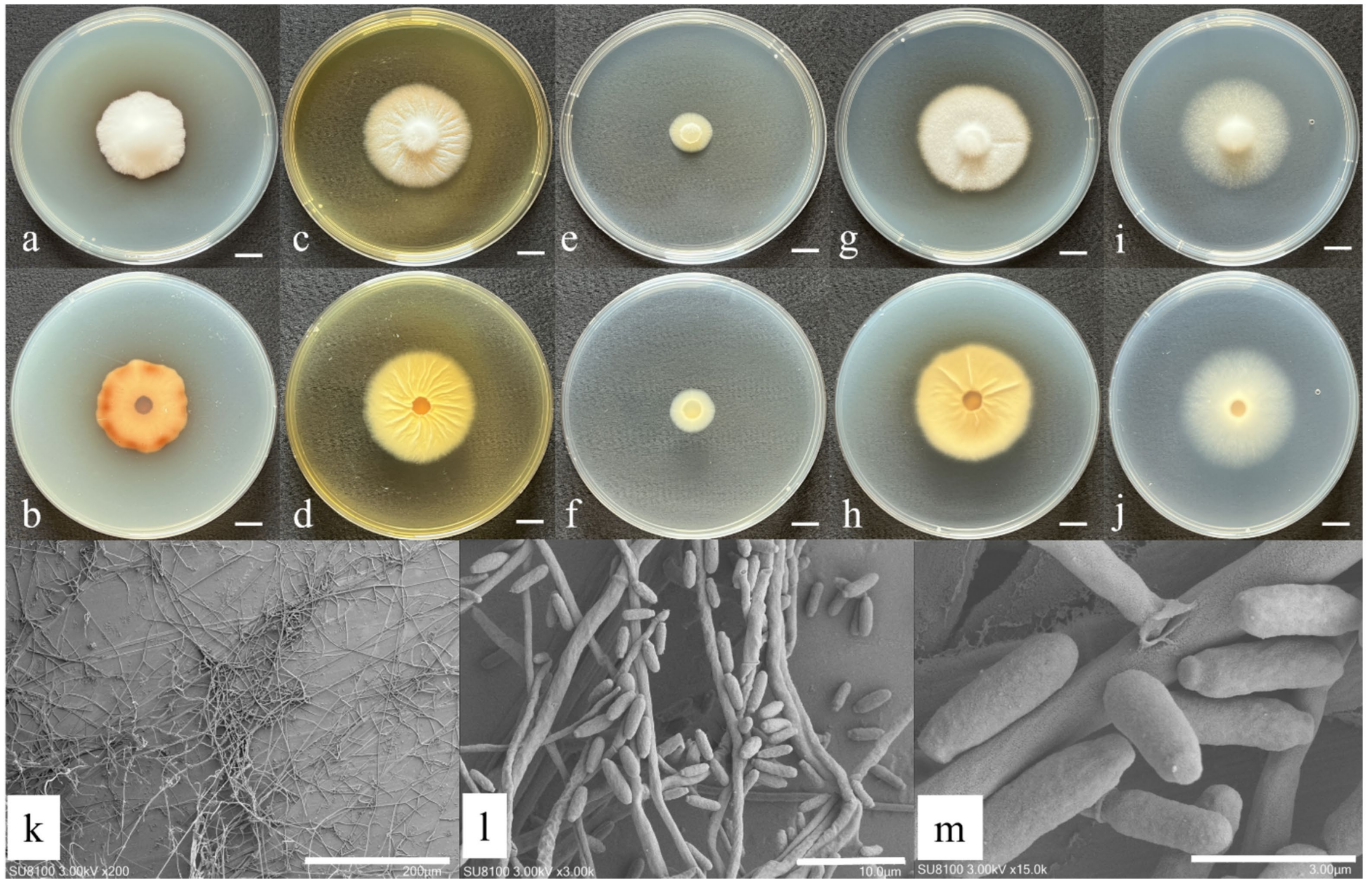
Morphological characteristics of *S. poyangense* sp. nov. **(a,b)** Colony morphology after 7 days of cultivation on PDA; **(c,d)** colony morphology after 7 days of cultivation on MEA; **(e,f)** colony morphology after 7 days of cultivation on DG18; **(g,h)** colony morphology after 7 days of cultivation on CYA; **(i,j)** colony morphology after 7 days of cultivation on Czapek; **(k,l)** sporulating structures and conidia; and **(m)** conidia. Scale: **(a–j)** = 1 cm.

**Figure 2:**
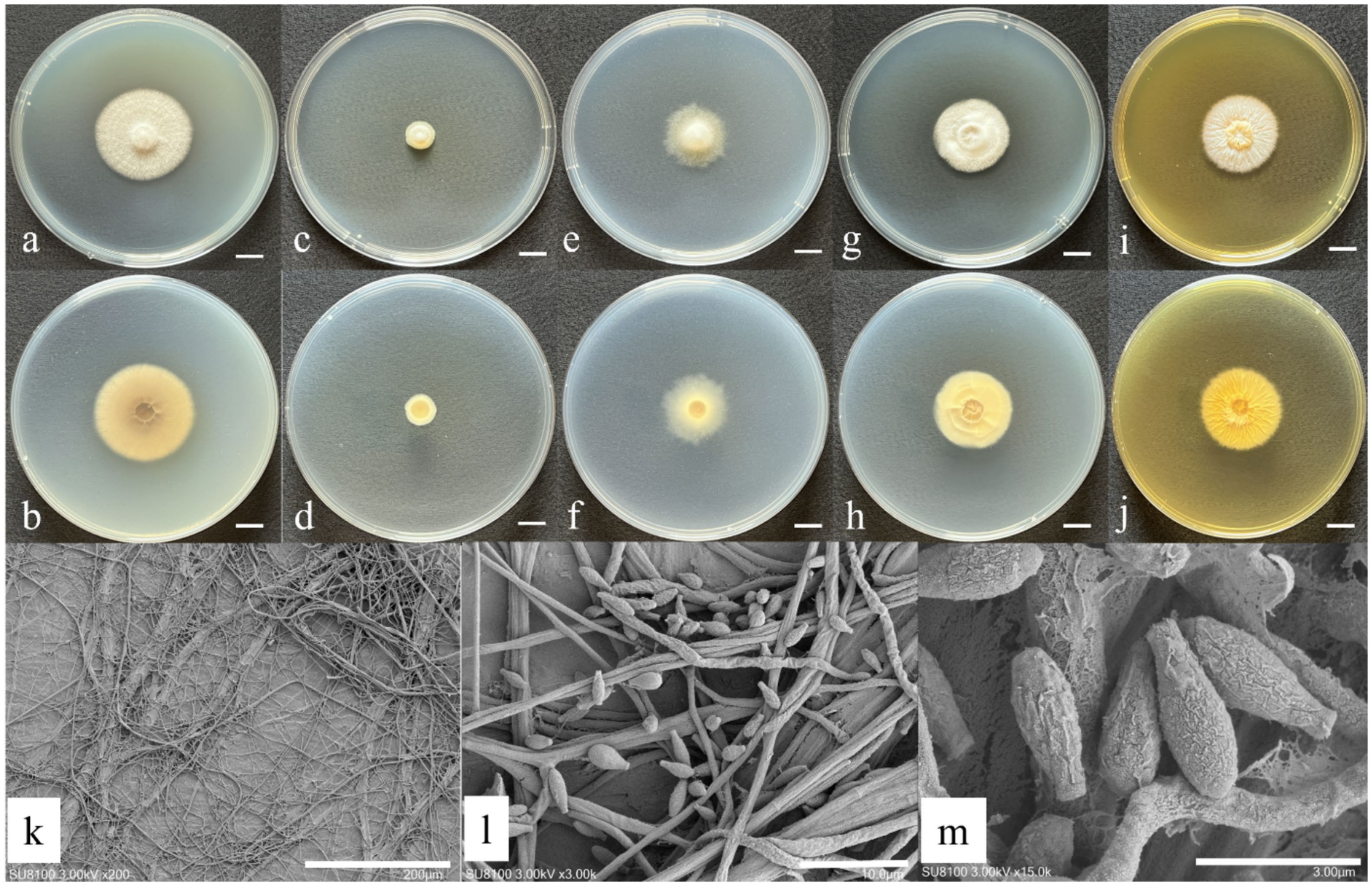
Morphological characteristics of *S. jiangsuense* sp. nov. **(a,b)** Colony morphology after 7 days of cultivation on PDA; **(c,d)** colony morphology after 7 days of cultivation on MEA; **(e,f)** colony morphology after 7 days of cultivation on DG18; **(g,h)** colony morphology after 7 days of cultivation on CYA; **(i,j)** colony morphology after 7 days of cultivation on Czapek; **(k,l)** sporulating structures and conidia; and **(m)** conidia. Scale: **(a–j)** = 1 cm.

The morphological characteristics and microscopic structures of the newly identified species cultured on various media are presented in Figures 1, 2. The detailed morphological description is as follows:

*Sarocladium poyangense* W. Chen and Q. Hu, sp. nov (Figure 1).

Index Fungorum Identifier: 905180.

Type: Isolated from fallow soil in Poyang County, Shangrao City, Jiangxi Province, China. Holotype specimen GDMCC 3.1075 (JX12E02) is a living specimen being maintained via lyophilization at Guangdong Microbial Culture Collection Center (GDMCC). The strain was collected by Wei Chen in June 2023.

Etymology: The species is named after its type locality, Poyang, Jiangxi Province, China.

Description: Hyphae septate, hyaline, thin- and smooth-walled, 1.55–2.52 μm wide, frequently aggregated forming net-like structures. Annelidic conidiogenesis, conidiophores erect to slightly flexuous, arising directly from vegetative hyphae, simple or sparingly branched, hyaline to subhyaline, 12.2–25.8 × 0.68–1.25 μm. Conidia unicellular, ellipsoid to cylindrical, 4.76–7.43 × 1.60–1.99 μm, hyaline to subhyaline, thin- and smooth-walled, often aggregated. Sexual morph not observed.

Cultural characteristics (after 7 days of incubation at 26 ± 1 °C in darkness): PDA: 35.67 ± 1.25 mm; obverse white, cottony-elevated; reverse rusty brown. CDA: 40.33 ± 0.47 mm; regularly circular; obverse beige, floccose; reverse pale yellow. CYA: 38.33 ± 0.47 mm; nearly circular; obverse beige, floccose; reverse pale yellow with distinct radial cracks. DG18: 15.67 ± 0.94 mm; slow-growing; obverse and reverse beige. MEA: 36.67 ± 0.94 mm; obverse pale yellow, floccose with distinct radial striations; reverse yellow.

Additional strains examined: ZJ19A01, was isolated from crop fields soil in Fuyang District, Hangzhou City, Zhejiang Province, China. The strain was collected by Wei Chen in June 2023.

Notes: Phylogenetic analyses revealed that *Sarocladium poyangensis* is closely related to *S. kiliense*, but they can be distinguished morphologically by their conidiogenesis. *S. poyangensis* produces conidia blastically, solitary or in opposite pairs, whereas conidia of *S. kiliense* are formed in clusters. In addition, *S. poyangensis* differs from *S. kiliense* by nucleotide differences in ITS (20 bp), nrLSU (5 bp), tef1 (15 bp) and RPB2 (67 bp).

*Sarocladium jiangsuense* W. Chen and Q. Hu, sp. nov (Figure 2).

Index Fungorum Identifier: 905181.

Type: Isolated from grassland soil in Danyang, Zhenjiang City, Jiangsu Province, China. Holotype specimen GDMCC 3.1076 (JS06C01) is a living specimen being maintained via lyophilization at Guangdong Microbial Culture Collection Center (GDMCC). The strain was collected by Wei Chen in June 2023.

Etymology: The species is named after its type locality, Jiangsu Province, China.

Description: Hyphae septate, hyaline, thin- and smooth-walled, 1.21–1.84 μm wide, frequently aggregated forming net-like structures. Annelidic conidiogenesis, conidiophores erect to slightly flexuous, arising directly from vegetative hyphae, simple or sparingly branched, hyaline to subhyaline, 12.28–32.34 × 0.58–1.89 μm. Conidia unicellular, lageniform to fusiform, 5.24–6.99 × 1.79–2.67 μm, hyaline to subhyaline, thin- and smooth-walled, often aggregated. Sexual morph not observed.

Cultural characteristics (after 7 days of incubation at 26 ± 1 °C in darkness): PDA: 31.67 ± 1.25 mm; obverse beige, cottony, concentric; reverse brown. CDA: 22.67 ± 0.94 mm; obverse yellow, floccose; reverse pale yellow. CYA: 27.33 ± 1.25 mm; obverse beige, floccose, concentric; reverse yellow with radial and concentric cracks. DG18: colonies small, 11.33 ± 0.47 mm; obverse white with concentric, reverse pale yellow. MEA: 26.67 ± 0.94 mm; obverse pale yellow with distinct radial cracks; reverse yellowish-brown. No sexual morph was observed on any medium.

Additional strains examined: JS07C02, was isolated from grasslands soil in Liyang City, Changzhou, Jiangsu Province, China. The strain was collected by Wei Chen in June 2023.

Notes: *Sarocladium jiangsuense* is closely related to *S. poyangensis*, but the two species can be clearly distinguished by their colony morphology. After 7 days of incubation on PDA, colonies of *S. jiangsuense* are beige and floccose on the surface, with brown reverse; whereas colonies of *S. poyangensis* are white and lanose on the surface, with rust-colored reverse. In addition, *S. jiangsuense* differs from *S. poyangensis* by nucleotide differences in ITS (26 bp), nrLSU (13 bp), tef1 (6 bp) and RPB2 (10 bp).

### 3.2 Phylogenetic analyses

Four *Sarocladium* sp. strains were isolated from soil samples. Phylogenetic analysis was conducted using a dataset comprising 49 strains representing 35 *Sarocladium* species retrieved from the NCBI database, along with the four strains identified in this study. *Chlamydocillium curvulum* CBS 430.66 was designated as the outgroup.

**Figure 3:**
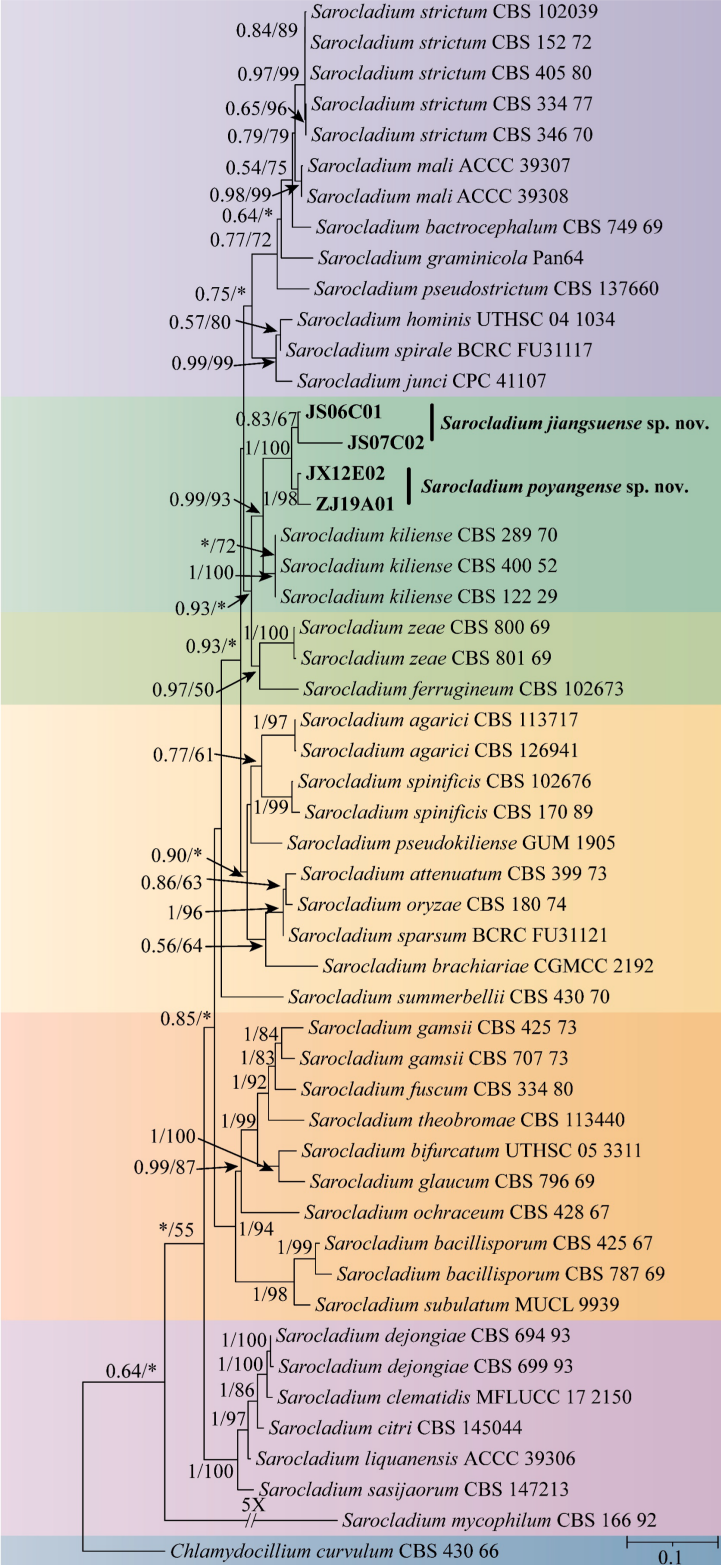
Phylogenetic tree of *Sarocladium* based on multiple gene loci (ITS, nrLSU, *act1, tef1*, and *RPB2*) combined with the maximum likelihood method. Bayesian posterior probability (BI ≥ 0.50) and IQtree bootstrap support values (ML ≥ 50) are labeled on the branch in the following order: BI/ML. BI < 0.50 and ML < 50 are marked as “*.” JS06C01 and JS07C02 are treated as conspecific despite the less-than-maximal support at their terminal node, as they form a distinct lineage and share consistent morphological and cultural characteristics.

In the Bayesian inference analysis, ModelFinder recommended the GTR + F + I + G4 model, while in the IQ-TREE analysis, the TIM2 + F + I + I + R3 model was selected. Phylogenetic analysis based on the concatenated dataset of five gene loci revealed that the four strains examined in this study clustered into a distinct clade, forming a highly supported independent lineage (BI/ML = 0.99/93) separate from the closely related species *S. kiliense*. Additionally, strains JS06C01 and JS07C02 as well as JX12E02 and ZJ19A01 each formed two highly supported subclades (BI/ML = 1/100), supporting their recognition as two novel species (Figure 3).

Based on morphological and phylogenetic analyses, *Sarocladium poyangense* is closely related to *S. kiliense*, but they can be distinguished by their sporulation patterns. Additionally, sequence divergence between *S. poyangense* and *S. kiliense* was observed in ITS, nrLSU, tef1, and RPB2 regions.

*S. jiangsuense* is closely related to *S. poyangense* but can be differentiated by colony morphology. Similarly, they also exhibit nucleotide differences in the four gene regions.

### 3.3 Biological activity of new species of *Sarocladium*

**Figure 4:**
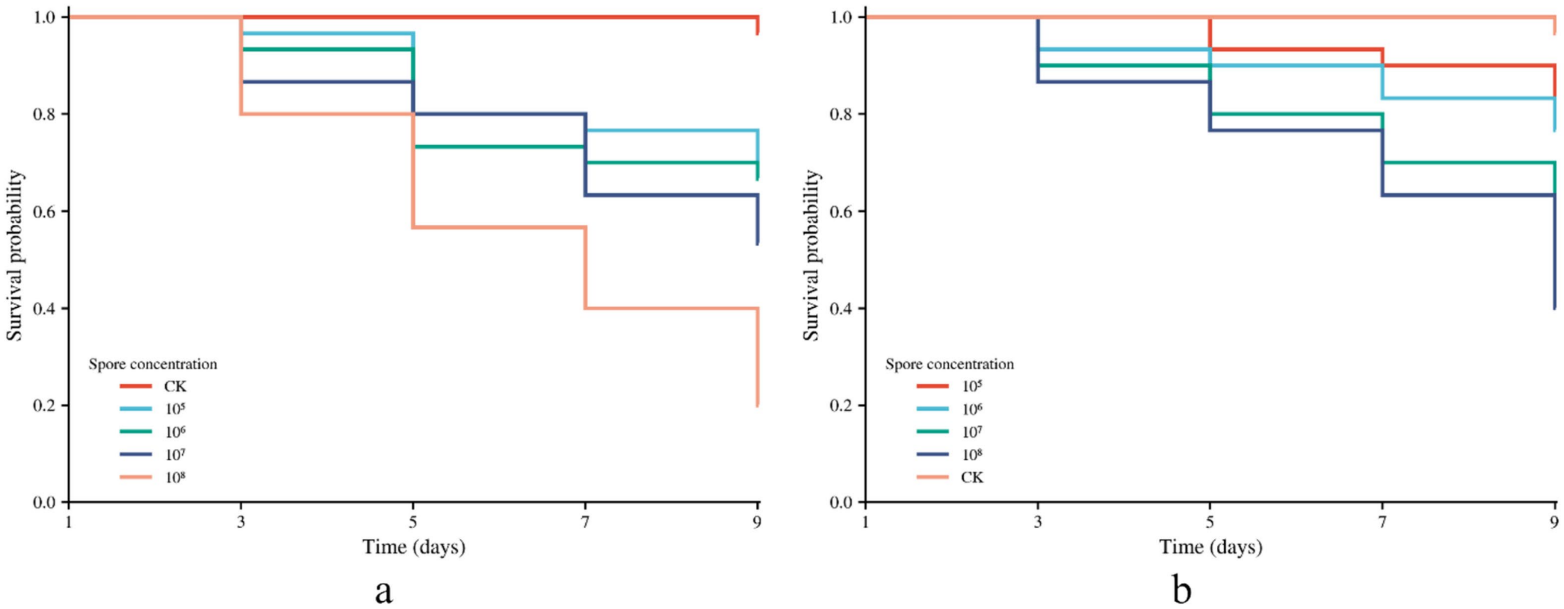
Biological activity of *Sarocladium* strains against the third-instar larvae of *O. furnacalis* at different concentrations: **(a)** JX12E02; **(b)** JS06C01. CK, 0.1% Tween-80 solution-treated larvae.

The bioactivity assay results for the two novel *Sarocladium* species against third-instar larvae of *O. furnacalis* are presented in Figure 4. The insecticidal activity of both strains showed a positive 256 correlation with spore concentration, with higher concentrations resulting in greater efficacy. At a spore concentration of 1 × 10^8^ spores/mL, *S. poyangense* strain JX12E02 achieved an 80% mortality rate against *O. furnacalis* by the ninth day.

The LC₅₀ and LT₅₀ values of the tested strains against *O. furnacalis* on the ninth day post-treatment at different spore concentrations are presented in Table 2. *Sarocladium poyangense* JX12E02 exhibited higher insecticidal activity, with an LC₅₀ value of 31.3 × 10^6^ spores/mL. In contrast, *S. jiangsuense* JS06C01 demonstrated relatively lower bioactivity. At a spore concentration of 1 × 10^8^ spores/mL, the LT₅₀ value of strain JS06C01 was 8.01 days, whereas strain JX12E02 exhibited a shorter LT₅₀ of 6.08 days.

**Table 2:** The LT_50_ of the third-instar larvae of *O. furnacalis* after infection with spore suspensions of different strains and concentrations and the LC_50_ on the ninth day.

Strain	LT_50_ (d Mean ± SE)			LC_50_ (9 d) (×10^6^)
	Concentration of conidia suspension(spores/mL)			
	1 × 10^8^	1 × 10^7^	1 × 10^6^	
JX12E02	6.08 ± 0.44	8.89 ± 0.72	-	31.3 ± 1.61
JS06C01	8.01 ± 0.44	-	-	71.9 ± 2.71

**Figure 5:**
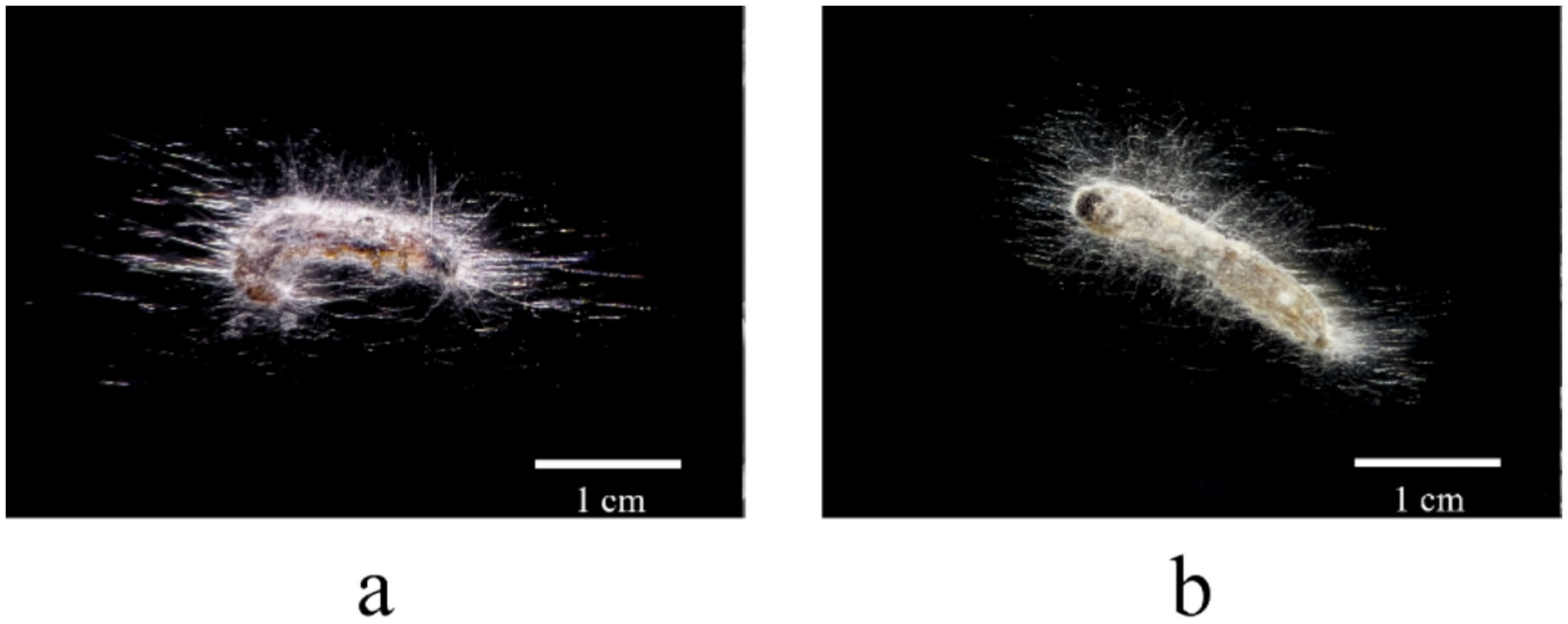
Morphological characteristics of fungal infection in insect: **(a)** JX12E02; **(b)** JS06C01.

The infection characteristics of the strains on the insect are illustrated in Figure 5. Microscopic observations revealed that white mycelia initially emerged from the abdomen, legs, and head of the infected insects. Over time, the mycelial growth progressively enveloped the entire body, with elongated hyphae extending beyond the cadaver. In the later stages of infection, grayish-white spore masses were observed surrounding the insect remains.

## 4 Discussion

This study successfully identified *S. poyangense* and *S. jiangsuense* as two novel species within the genus *Sarocladium*, supported by multigene phylogenetic analyses. These species form distinct evolutionary lineages, separate from their closest relative, *S. kiliense*. Morphological characterization further corroborates their taxonomic independence; *S. poyangense* produces conidia by budding, either singly or in pairs, whereas *S. kiliense*, which forms conidial clusters. Additionally, colony morphology on PDA differs significantly, with *S. poyangense* exhibiting a white, cottony texture with a rust-colored reverse, while *S. jiangsuense* presents a pale yellow, velvety surface with a brown reverse. These distinct morphological and phylogenetic features provide robust evidence supporting the recognition of *S. poyangense* and *S. jiangsuense* as novel species within the genus *Sarocladium*.

This study reports the first entomopathogenic activity in the genus *Sarocladium*. Previous research has primarily associated *Sarocladium* species with plant endophytism and phytopathogenicity, including their roles in rice sheath rot (*S. oryzae*) and bamboo blight (*S. strictum*; Zhang et al., 2022). However, no prior studies have documented insecticidal properties within this genus. Our bioassay results demonstrate that *S. poyangense* and *S. jiangsuense* exhibit significant pathogenicity against third-instar larvae of *O. furnacalis*, with mortality rates positively correlated with conidial concentration. Notably, *S. poyangense* JX12E02 achieved an 80% mortality rate at 1 × 10^8^ spores/mL by the ninth day post inoculation, highlighting its potential as an effective entomopathogen.

Beyond the Koch’s, already fulfilled in this study, several additional criteria remain important to strengthen the conclusion that *Sarocladium* represents a bona fide entomopathogenic lineage. Documenting typical post-mortem mycosis and providing time-course microscopic evidence of key steps in the infection would directly demonstrate an infection-driven process. In parallel, separating infection effects from toxin-only effects by including heat-killed conidia and cell-free culture filtrate controls, together with reporting inoculum quality and the environmental envelope for infection, would reduce confounding and allow the entomopathogenic activity to be interpreted with greater confidence.

Quantitative assessments further substantiate the insecticidal efficacy of these species. The LC₅₀ for *S. poyangense* was determined to be 31.3 × 10^6^ spores/mL, indicating higher virulence than *S. jiangsuense*. Furthermore, at a conidial concentration of 1 × 10^8^ spores/mL, the LT₅₀ for *S. poyangense* was 6.08 days, significantly shorter than the 8.01 days observed for *S. jiangsuense*. These findings suggest that *S. poyangense* exhibits superior insecticidal efficacy and warrants further investigation as a potential biocontrol agent.

Although the precise mode of action remains to be elucidated, our findings suggest that the insecticidal activity of *S. poyangense* and *S. jiangsuense* may involve a combination of mechanical disruption of the cuticle and the production of insecticidal metabolites. Similar mechanisms have been reported in well-characterized entomopathogenic fungi such as *Metarhizium* spp. and *Beauveria* spp., in which host invasion is facilitated by cuticle-degrading enzymes and the secretion of secondary metabolites with insecticidal properties (Golzan et al., 2023; Shang et al., 2024). Future research should focus on identifying the virulence factors and secondary metabolites that contribute to the pathogenicity of these *Sarocladium* species.

Given their demonstrated entomopathogenic activity, *S. poyangense* and *S. jiangsuense* hold promise as novel biocontrol agents against *O. furnacalis*. Compared with chemical insecticides, microbial control agents offer a more sustainable, environmentally friendly option for pest management (Bravo and Soberón, 2023). Nevertheless, several issues must be addressed before these fungi can be considered for practical application. This study focused exclusively on *O. furnacalis*; future work should evaluate efficacy across a broader range of agriculturally relevant pests to clarify host range and potential specificity. Scalable production and formulation require optimization of culture conditions to maximize sporulation and preserve virulence, and laboratory performance must ultimately be validated in field settings where environmental variability can strongly influence infection success. Potential non-target effects and ecological risks should be examined systematically to ensure biosafety and regulatory readiness.

A further ecological consideration is that *Sarocladium* species are frequently isolated from plant tissues and have been reported as endophytes or as agents associated with plant diseases, indicating that plant association may be common within the genus (Gama et al., 2020). However, endophytic colonization and any consequent plant effects are functional traits that cannot be inferred solely from taxonomy or insect bioassays. The present study was designed to delimit species boundaries and evaluate entomopathogenicity under standardized insect bioassays, we did not test plant colonization or plant performance responses for *S. poyangense* and *S. jiangsuense*. Accordingly, their endophytic capacity and impacts on plants remain unknown based on the current dataset. Notably, dual ecological roles—plant association together with insect pathogenicity—are biologically plausible and have been described in other fungal lineages, suggesting that these new species may have broader functional potential than direct insect control alone (Moraga, 2020). Future studies should therefore assess whether *S. poyangense* and *S. jiangsuense* can colonize crop plants as endophytes and confer measurable agronomic effects, such as growth promotion, induced resistance to pests and diseases, or enhanced tolerance to abiotic stress.

While this study provides the first evidence of insecticidal activity in the genus *Sarocladium*, several limitations should be acknowledged. The molecular mechanisms underlying the entomopathogenicity of *S. poyangense* and *S. jiangsuense* remain unclear. Whole-genome and transcriptomic analyses could facilitate the identification of key virulence genes and secondary metabolite biosynthetic pathways. Additionally, the host range of these fungi is currently unknown. Comparative pathogenicity assays on diverse insect species will be necessary to determine whether their entomopathogenic activity is host-specific or broadly effective. Finally, this study was conducted under controlled laboratory conditions; future field trials will be essential to validate the biocontrol potential of these fungi in agricultural ecosystems.

Overall, our findings not only expand the taxonomic diversity of *Sarocladium* but also provide novel insights into its ecological roles, particularly its potential as a microbial control agent. Further research is warranted to elucidate the molecular basis of its entomopathogenicity and to evaluate its feasibility for large-scale biocontrol applications.

## 5 Conclusion

This study reports the first insecticidal activity in the genus *Sarocladium*, identifying *S. poyangense* and *S. jiangsuense* as novel entomopathogenic fungi. *Sarocladium poyangense* exhibited greater virulence against *O. furnacalis*, as indicated by its lower LC₅₀ and shorter LT₅₀ compared to *S. jiangsuense*. These findings extend the known ecological roles of *Sarocladium*, highlighting its potential for application in biological control strategies. Future research should focus on elucidating the molecular mechanisms underlying its entomopathogenicity, optimizing mass production techniques, and conducting field trials to assess its efficacy within integrated pest management programs.

## Data Availability

The datasets presented in this study can be found in online repositories. The names of the repository/repositories and accession number(s) can be found here: https://www.ncbi.nlm.nih.gov/genbank/, PP863315, PP930916, PV294978, PV294977, PV294976; PP863314, PV248877, PV294981, PV294980, PV294979; PP863316, PP930917, PV294972, PV294971, PV294970; PP863317, PV248878, PV294975, PV294974, PV294973.
